# Natural elicitors enhanced suberin polyphenolic accumulation in wounded potato tuber tissues

**DOI:** 10.3389/fpls.2024.1384602

**Published:** 2024-05-28

**Authors:** Munevver Dogramaci, Dipayan Sarkar, Fernando L. Finger, Kalidas Shetty, Karen K. Fugate

**Affiliations:** ^1^ Edward T. Schafer Agricultural Research Center, United States Department of Agriculture (USDA)-Agricultural Research Service, Fargo, ND, United States; ^2^ Universidade Federal de Viçosa, Centro de Ciências Agrárias, Departamento de Agronomia, Av. P.H. Rolfs, Viçosa, MG, Brazil; ^3^ Department of Plant Sciences, North Dakota State University, Fargo, ND, United States

**Keywords:** antioxidant, elicitors, phenolic metabolites, stress response, suberin polyphenolics, wound-healing

## Abstract

**Introduction:**

Unintended wounding or bruising during harvest or postharvest handling leads to significant tuber loss and imposes economic burden to potato industry. Therefore, finding effective strategies to mitigate wound-related tuber losses is very important from industry perspectives. Formation of protective barrier through accumulation of suberin polyphenolics (SPP) is a natural and initial response of potato tuber tissues to wounding.

**Materials and methods:**

In this study, efficacy of two natural elicitors, such as chitosan oligosaccharide (COS 0.125 g L^-1^) and cranberry pomace residue (Nutri-Cran 0.125 g L^-1^) was investigated using a mechanically wounded tuber tissue model and by histological determination of SPP formation in five agronomically relevant and red-skin potato cultivars (Chieftain, Dakota Rose, Dakota Ruby, Red LaSoda, Red Norland). Furthermore, the potential role of stress protective metabolic regulation involving phenolic metabolites, proline, and antioxidant enzymes in tuber WH processes were also investigated during 0-9 days after wounding.

**Results and discussion:**

Exogenous treatments of both COS and Nutri-Cran resulted into enhanced SPP formation in wounded surface, but the impact was more rapid with Nutri-Cran treatment in select cultivars. Greater contents of total soluble phenolic, ferulic acid, chlorogenic acid, total antioxidant activity, and superoxide dismutase activity were also observed in elicitor treated tuber tissues at different time points after wounding. Nutri-Cran treatment also reduced the activity of succinate dehydrogenase in Red Norland and Dakota Ruby at 3 d, indicating a suppression in respiration rate. Collectively, these results suggest that Nutri-Cran can be potentially utilized as an effective WH treatment to potato tubers for minimizing wound-related losses.

## Introduction

Potato (*Solanum tuberosum* L.) is grown as a major staple food crop in about 125 countries around the world (CIP, 2021), and supports basic calorie needs of almost one billion people as rich source of resistant starch, vitamin C, essential minerals, and fibers. Protecting nutritional qualities of potato tubers after harvest and enhancing their shelf-life in storage is critical for sustaining global food supply chain and to address nutritional insecurities (Devaux et al., 2021). One of the widespread challenges that growers and potato industry face is significant loss of harvested tubers due to wounding or bruising related damages (10-15%). Potato tubers can be damaged during harvesting, post-harvest handling, packing, and transporting, which leads to the exposure of wounded bare tissues to different kinds of biological and environmental stresses (Wahrenburg et al., 2021). Cutting seed tubers in several pieces, which is part of the common pre-planting practice in potato growing regions also induces wound-related losses in the field after planting. All types of wounding (bruising, cutting, skinning) of potato tubers cause water loss, texture (skin) aberrations, untimely sprouting, and increase susceptibility to bacterial and fungal diseases (Lulai, 2007). Such deterioration in the texture and quality of potato tubers affects their retail value, leading to poor economic returns for potato industry stakeholders. Therefore, it is important to enhance healing of wounded potato tubers for protecting postharvest qualities and shelf-life and to mitigate decay of cut seed tuber pieces before planting.

Like any other viable biological tissues, potato tubers have natural wound healing (WH) mechanism, which involves formation of protective barrier in the wounded surface. Once the protective barrier, which is also known as suberin barrier, is formed, it minimizes water vapor loss, and protect tubers from pathogen infection and damages by insects. Furthermore, wounded tubers or cut seed tuber pieces with fully formed suberin layer are generally resilient against any environmental stresses and physical decay. Overall, tuber WH processes can be divided into two distinct phases, the immediate response is known as i) closing layer formation, which is followed by ii) wound periderm formation (Lulai and Neubauer, 2014; Singh et al., 2021). Accumulation of suberin polyphenolics (SPP) and suberin polyaliphatics (SPA) is an intrinsic response of tuber tissues to wounding and are major steps of the two stage WH process (Lulai et al., 2008; Lulai and Suttle, 2009). In cell wall of wounded tissues, SPP, which is comprised off hydroxy-cinnamic acid and its derivatives, form a lignin-like structure (Lulai, 2007). Without formation of SPP, SPA accumulation does not occur in the polyphenolic matrix. Therefore, accumulation of SPP is the major and initial indicator of the WH responses of potato tuber tissues (Lulai and Neubauer, 2014; Lulai et al., 2016).

Strategies to improve WH process of potato tubers needs to promote the rapid accumulation of SPP in the wounded tissue surface. Currently, no practical postharvest methodologies are available to accelerate rapid accumulation of SPP and formation of suberin barrier in wounded or bruised tubers. Previous studies have highlighted positive impacts of exogenous hormone and elicitor treatments, such as brassinosteroids (BRs) (Han et al., 2022a), abscisic acid (ABA) (Li et al., 2017), jasmonic acid (JA) (Ozeretskovskaya et al., 2009), methyl jasmonate (Zhou et al., 2019), salicylic-acid analogue (Jiang et al., 2022), nitric oxide (Han et al., 2020; Dogramaci et al., 2024), and sodium silicate (Han et al., 2022) on WH responses of potato tuber tissues. All these studies observed formation of SPP and lignin in wounded surface of potato tubers during initial WH process. Furthermore, these studies indicated that induction of natural stress responses involving reactive oxygen species (ROS), phenylpropanoid metabolites, phytohormones (ABA, ethylene, JA), and fatty acids are associated with WH processes of potato tubers (Kumar et al., 2010; Lulai and Neubauer, 2014; Lulai et al., 2016; Zheng et al., 2020; Wahrenburg et al., 2021). However, these studies have not yet led to find a practical solution that could be integrated in commercial storage or pre-planting practices for mitigating wound related tuber losses. Therefore, it is essential to continue the search for finding safe and effective strategies for improving WH processes of potato tubers.

Previously, Chintha et al. (2023) observed that natural elicitors such as water-soluble chitosan oligosaccharide (COS) and cranberry pomace residue (Nutri-Cran) have positive impacts on SPP accumulation and overall WH processes in mechanically wounded tissues of potato tubers (cvs. Russet Burbank and Russet Norkotah). They also observed that elicitor treatments induced natural stress responses involving pentose phosphate pathway (PPP) regulation and proline metabolism and enhanced stress protective phenolic metabolite content and antioxidant activities in wounded tissues. Plant’s first line of defense response against wound or other abiotic stresses involve oxidative PPP, which provides precursors for biosynthesis of amino acids and nucleotides (Tisi et al., 2008). Regulation of PPP is also important for the generation of reducing molecules such as NADPH, which is a major electron donor and contributes to counter wounding-induced oxidative pressure. Therefore, diversion of carbon flow from glycolysis to PPP favors tissue and cellular repair and helps to maintain redox homeostasis. It is also proposed that synthesis of proline in the cytosol is coupled with PPP through NADP^+^/NADPH regulation and proline metabolism is integral part of plant’s defense response (Shetty and Wahlqvist, 2004). Overall, elicitors derived from natural sources induce defense responses in plant cells and provide protection against abiotic and biological stresses.

Chitosan and its derivatives are bioactive polymeric compounds with diverse biological functions and have shown promises as effective WH treatment in both plant and animal models (Hu et al., 2020; Moeini et al., 2020; Li et al., 2021; Prasathkumar and Sadhasivam, 2021). Spraying of chitosan during fruit development has resulted into rapid deposition of SPP in artificially wounded sites of muskmelon (*Cucumis melo* L.) after harvest (Li et al., 2021). The same study also reported activation of stress protective phenylpropanoid pathway with chitosan treatment (Li et al., 2021). Extended shelf-life of fresh cut paprika (*Capsicum annum* L. var. *grossum*) and enhanced antioxidant activity were noticed with chitosan nanoparticle coating (Hu et al., 2020). Similarly, enhanced biosynthesis of phenolic metabolites and lignin content was observed in wounded apple (*Malus domestica* Borkh. cv. Fuji) tissues with chitosan dipping treatment (Ackah et al., 2022). Collectively, these studies indicated that chitosan induces stress protective and secondary metabolic responses and improve WH of plant tissues. Cranberry pomace residues are also rich sources of phenolic metabolites and natural antioxidants and have distinct bioactive properties relevant for WH applications and protection against pathogens (Côté et al., 2010; Tamkutė et al., 2019).

However, the impact of chitosan and cranberry pomace residues on WH responses of potato tubers and their potential mechanisms were not examined thoroughly. Furthermore, the effect of these natural elicitors on potato WH responses may vary among different potato cultivars. Therefore, the main objective of this study was to investigate the efficacy of COS and Nutri-Cran treatments on WH processes of five potato cultivars known to have contrasting skin-set trait. The formation of SPP in wounded site of tuber tissues along with profile of phenolic metabolites were determined during the closing layer formation (0-9 days after wounding). Additionally, the stress response related metabolic regulations involving oxidative PPP, tricarboxylic acid cycle (TCA), proline metabolism, and antioxidant enzyme activities were also monitored in wounded tuber tissues with and without natural elicitor treatments. The aim of this study was not only to determine the efficacy of COS and Nutri-Cran treatments for improving WH responses of tubers, but also to elucidate their impact on tuber WH mechanisms of different potato cultivars.

## 2 Materials and methods

### 2.1 Potato tubers

Seed tubers of five red-skin cultivars (Chieftain, Dakota Rose, Dakota Ruby, Red LaSoda, Red Norland), with contrasting skin-set trait were originally collected from a commercial grower. Seed tubers were then grown in the Northern Crop Science Laboratory greenhouse (USDA, Fargo, USA) under optimum growing conditions (16/8 h light/dark, temperature range of 21-24°C), and matured tubers were harvested and used for this experiment. After harvest tubers were allowed to be conditioned for skin set at 20°C for two weeks at 90-95% relative humidity (RH) in an environmental chamber (Conviron GEN1000-GE, Winnipeg, MB, Canada). After two weeks, the temperature was ramped down from 20°C to 8°C (~1.5°C/day) and kept at 8°C. Prior to the WH experiment, tubers were moved from the chamber and washed with distilled water and equilibrated at 20°C and ~95% RH for 72 h.

### 2.2 Wounding of potato tubers and application of elicitor treatments

A tuber disc model system as described by Lulai et al. (2008) was adapted to study the WH responses of potato tubers. In brief, a cork borer was used to laterally excise a cylinder (11 mm diameter) of tuber flesh tissues, and discs of parenchyma tissues were cut using a fixed width blade (3 mm thick) as part of mechanical wounding model.

**Figure 1:**
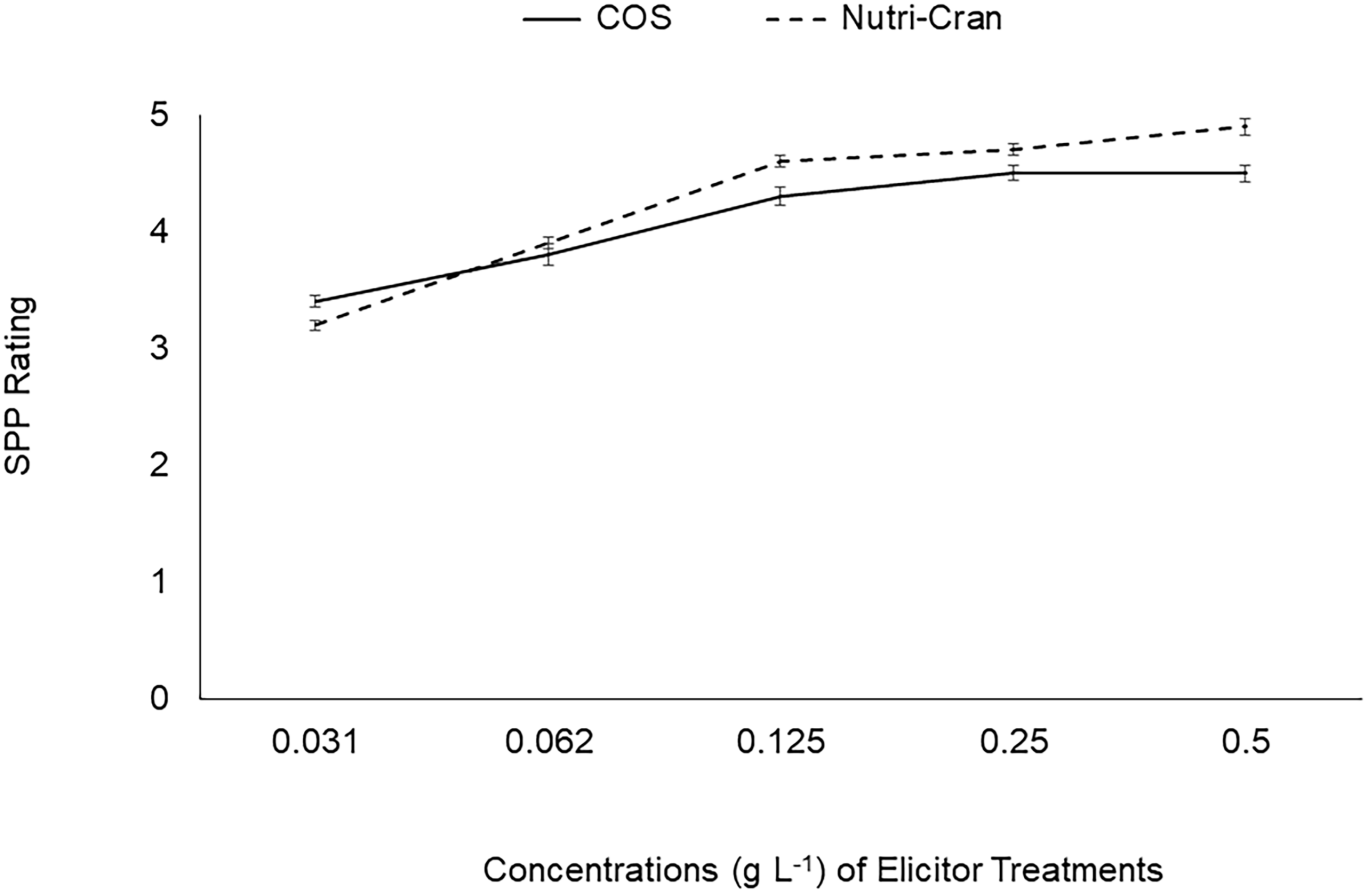
Changes in SPP rating with different concentrations (0.031, 0.062, 0.125, 0.250, 0.500 g L^-1^) of chitosan oligosaccharide (COS) and cranberry pomace extract (Nutri-Cran) at 4 days after wounding of tuber tissues.

Immediately after wounding, tuber discs were treated with two elicitor treatments [water soluble chitosan oligosaccharide with an ascorbic acid sidechain (COS; Kong Poong Bio, Jeju, South Korea) and bioprocessed cranberry pomace residue (Nutricran; Decas Botanical Synergies, Wareham, MA, USA)] at 0.125 g L^-1^ rate. Elicitors were dissolved in 0.01 M MES (2-(N-morpholino) ethanesulfonic acid) buffer (pH 5.7), and MES alone was used as the control treatment. Additionally, 0.1 mM fluridone (FL), an ABA biosynthesis inhibitor, which slows down SPP formation was also used as a treatment to compare with the effect of the elicitor treatments. Concentration (0.125 g L^-1^) of elicitor treatments was selected based on the initial optimization with dose dependent response of elicitor treatments on SPP formation at 4 d after wounding (**Figure 1**). Tuber discs were placed in the respective elicitor and control treatment solutions (60 discs in 100 mL) and allowed to imbibe for 1 h on a rotary shaker (Innova 2000, New Brunswick Scientific, Edison, NJ, USA) (50 cycles min^-1^). Treatment solutions were replaced with fresh solution every 20 min. Then, treated tuber discs were transferred and kept as a single layer on a non-reacting mesh surface in Pyrex trays and allowed to wound-heal in the dark at 20°C and ~95% RH. A subset of discs was snap frozen in liquid nitrogen at 0 h and remaining discs were collected at different time points (3, 6, and 9 d after wounding) for determination of phenolic and proline content and to conduct biochemical enzyme analysis. Collected tuber discs were kept at -80°C freezer.

### 2.3 Histological determination of suberin polyphenolics accumulation in wounded surface of potato tuber tissues

A subset of discs (4 discs per biological replicate and total 4 replicates) from 3, 6, and 9 d after the elicitor treatments were placed in Farmer’s fixative (absolute ethanol: acetic acid in 3:1 v/v) for microscopical analysis of SPP. A microscope (Zeiss Axioscope) configured with fluorescent illumination was used to determine the suberization rating based on the SPP accumulation at the wound-healed surface of the tuber discs. The suberization rating system developed by Lulai and Corsini (1998) was employed which indicated 0= none; 3.0= contiguous accumulation of SPP on outer tangential layer of first cell, 5= complete accumulation of SPP around first cell layer, 7= complete accumulation around first and second cell layers, and 9= expanded accumulation towards third parenchymal cell layer.

### 2.4 Tissue extraction for biochemical analysis

Subset of frozen tuber tissues from each time point (0, 3, 6, 9 d) were ground into a fine powder using Qiagen Tissue Lyser for 30 s (Retsch.Lab Equipment, Newtown, PA, USA). Following grinding, frozen potato tissue powder were weigh and kept in 2 mL Eppendorf centrifuge tubes and stored at -80°C.

A subset of ground tissue samples (100 mg) was transferred to glass vials and 95% ethanol (5 mL) was added. All vials were then stored at 4°C for 48 h. A tissue homogenizer (BioSpec Products Inc., Bartlesville, OK, USA) was used to homogenize the tuber tissues and the homogenate was centrifuged (10,000 g; 5 min). Collected supernatant were used to determine total soluble phenolic (TSP) content, total antioxidant activity, and phenolic profile of the tuber tissues.

For all enzyme analysis and determination of protein and proline content, ground potato tissue (400 mg) was transferred in a chilled pestle and mortar and mixed with 4 mL enzyme extraction buffer (0.5% polyvinylpyrrolidone (PVP), 0.003 M EDTA (Ethylenediaminetetraacetic acid), and 0.1 M potassium phosphate buffer; pH 7.5). Following maceration, potato tissue homogenates were centrifuged at 10,000 g for 10 min. Supernatant were collected and used for the enzyme analysis.

### 2.5 Determination of TSP content, phenolic profile, proline content, and total protein content

A colorimetric assay protocol originally described by Shetty et al. (1995) was used to determine TSP content. Supernatant (50 mL) were transferred to a glass test tube and were mixed with 1 mL 95% ethanol, 5 mL distilled water, 0.5 mL Folin-Ciocalteu reagent and 1 mL Na_2_CO_3_. A vortex shaker was used to mix the reagents thoroughly and the mixture was incubated in the dark for 1 h. Following incubation, absorbance of the mixture was recorded at 725 nm (Thermo Fisher Evolution Series, MA, USA). A standard curve using different concentrations of gallic acid (0.01 – 0.30 g L^-1^) was prepared, and TSP content of the sample was determined and expressed as mg g^-1^ fresh weight (FW).

Phenolic acid profile was characterized using a reverse phase high performance liquid chromatography-(HPLC) method [Agilent 1260 Infinity Series equipped with DAD 1100 diode array detector (Agilent Technologies, Palo Alto, CA, USA)]. A step gradient elution protocol was used with the following solvents: 0.01 M phosphoric acid (pH 2.5; Solvent A) and 100% methanol (Solvent B). For first 8 min, the methanol concentration was increased to 60%, then increased to 100% over next 7 min, then decreased to 0% for next 3 min, and was maintained at 0% for 7 min with total run time of 25 min. A C-18 analytical column (Agilent Supelco SB-C18 250 x 0.046 m internal diameter) with a packing material particle size of 0.05 mm was used and eluted at a flow rate of 0.7 mL min^-1^ at ambient temperature. Chromatograms were recorded at 214, 230, 260, and 306 nm wavelength. Pure standards of gallic acid, catechin, chlorogenic acid, ferulic acid, caffeic acid, and benzoic acid in 100% methanol were used to calibrate retention times and to create the standard curve. An Agilent Chemstation integration software was used to determine the concentration of individual phenolic compounds, which was expressed as µg g^-1^FW.

A liquid chromatography-based protocol (Chintha et al., 2023) using an Agilent 1260 Infinity Series equipped with DAD 1100 diode array detector (Agilent Technologies, Palo Alto, CA, USA) was used to determine the total proline content. A reverse phase Eclipse C18 column with 0.25 x 0.0046 m internal diameter and packing material of 0.005 mm particle size was used. An isocratic elution using 20 mmol potassium phosphate buffer (pH 2.5 by phosphoric acid) at a flow rate of 1 mL min^-1^ was used and the chromatogram was obtained at 210 nm. A standard curve was prepared using different concentrations of L-Proline, which is dissolved in 20 mmol potassium phosphate solution. The amount of proline in the potato tissue sample was expressed as mg g^-1^ FW.

The total soluble protein content of the potato tissue extracts was determined using Bradford protein binding assay (Bradford, 1976). Bio-Rad dye reagent (Bio-Rad Laboratories, Hercules, CA, USA) was diluted with distilled water (1:4 ratio) and 5 mL solution was mixed with potato tissue extracts. The mixture was then incubated in the dark for 5 min and its absorbance was measured at 595 nm. A blank using distilled water instead of sample was used. Standard solutions of bovine serum albumin were dissolved in distilled water to prepare a standard curve, based on which the soluble protein content (mg g^-1^) of the sample extracts was calculated.

### 2.6 Determination of glucose-6-phosphate dehydrogenase, succinate dehydrogenase, and proline dehydrogenase activities

A colorimetric method originally described by Deutsch (1978) was used to determine the G6PDH activity of potato tissue extracts. An enzyme reaction mixture containing ß-NADP (0.0058 mM Nicotinamide adenine dinucleotide phosphate), MgCl_2_ (0.088 mM magnesium chloride), glucose-6-phosphate (0.053 mM), and maleimide (0.77 mM) was prepared. A baseline (zero) was set at 340 nm using the reaction mixture. Then reaction mixture (1 mL) was mixed with 100 μL potato tissue extracts and the shift in absorbance over 5 min was used to quantify G6PDH activity in the samples based on the extinction coefficient of NADPH (reduced nicotinamide adenine dinucleotide phosphate), (0.0622 mM^-1^ m^-1^).

To determine SDH activity, an assay protocol described by Bregman (1987) was used. A reaction mixture containing 0.5 mL potassium phosphate buffer (0.4 M; pH 7.2), 0.2 mL sodium succinate (0.15 M; pH 7.0), 0.2 mL sodium azide (0.2 M), and 0.0001 L 2,6-dichlorophenolindophenol (DCPIP; 6.0 g L^-1^) was prepared. The baseline was established using the enzyme extraction buffer solution at 600 nm wavelength. Then reaction mixture (1 mL) was added to 200 μL potato tissue extracts and the rate of change of absorbance per min was measured to quantify SDH activity in the sample, based on the extinction coefficient of DCPIP (0.0191 mM^-1^ m^-1^).

A method described by Costilow and Cooper (1978) was used to determine the activity of PDH. The enzyme reaction mixture containing 100 mM sodium carbonate buffer (pH 10.3), 20 mM L-proline solution and 10 mM NAD (Nicotinamide adenine dinucleotide), was prepared and used for the assay. In the 1 mL reaction mixture, 200 μL potato tissue extracts were added and the increase in absorbance was measured at 340 nm over an interval of 3 min at 32°C. One unit of PDH activity was defined as amount required to cause a shift of 0.01 absorbance units per min at 340 nm (0.01 m light path).

### 2.7 Determination of total antioxidant activity and activities of antioxidant enzymes (catalase-CAT, guaiacol peroxidase-GPX, and superoxide dismutase)

Total antioxidant activity was measured using the ABTS [2, 2' –azinobis (3-ethylbenzothiazoline-6-sulfonic acid)]-based radical cation decolorization assay (Re et al., 1999). One day prior to conducting the assay, a stock solution was prepared by mixing 0.007 M ABTS solution with 0.14 M K_2_S_2_O_4_ solution. The mixed solution was kept in the dark at 4°C for maturation. After maturation, 95% ethanol was added to prepare a working ABTS solution with an absorbance of 0.70 ± 0.02 units at 734 nm. The potato tissue extracts were mixed with ABTS stock solution in a micro centrifuge tube and mixed thoroughly using a vortex mixer. The mixture was then incubated at room temperature for 2.5 min and absorbance was recorded at 734 nm. The antioxidant activity of the extracts was expressed as percentage (%) inhibition of ABTS radical formation and was calculated per the following formula:


Inhibition (%)=(Abs_control−Abs_sample)/Abs_control×100

A colorimetric method described by Beers and Sizer (1952) was used to determine CAT activity. A reaction mixture containing 0.059 M hydrogen peroxide (Merck’s Superoxol or equivalent grade, Merck Co. & Inc., Whitehouse Station, NJ) and 0.05 M potassium phosphate buffer solution (pH 7.0) was prepared. This mixture was incubated in a spectrophotometer for 4–5 min to achieve temperature equilibration and to establish a baseline. In 1450 μL the reaction mixture, 50 μL of potato tissue extracts was added and the disappearance of peroxide due to activity of CAT was monitored by measuring the shift in absorbance at 240 nm in 1 min. The change in absorbance from the initial linear portion of the curve was calculated. One unit of catalase activity was defined as amount that decomposes one micromole of H_2_O_2_.

The GPX activity was determined using a method described by Laloue et al. (1997). A reaction mixture was prepared by mixing 0.1 M potassium phosphate buffer (pH 6.8), 56 mM guaiacol solution, and 50 mM hydrogen peroxide (H_2_O_2_). Then 990 μL of reaction mixture was mixed with 10 μL potato tissue extracts and the shift in absorbance over 3 min was measured. Based on the extinction coefficient of the oxidized product tetraguaiacol (0.266 mM^-1^ m^-1^), GPX activity of the sample was determined.

Activity of SOD was determined using a colorimetric method described by Oberley and Spitz (1984). The reduction of nitroblue tetrazolium (NBT) by sample extracts at 560 nm was monitored. The reaction mixture was prepared by mixing 13.8 mL of 50 mM potassium phosphate buffer (pH 7.8) containing 1.33 mM diethylene tetra amine penta acetic acid; 2.45 mM NBT (0.5 mL); 1.8 mM xanthine (1.7 mL) and catalase (40000 IU L^-1^). Then in the reaction mixture (0.8 mL), phosphate buffer (0.1 mL) and xanthine oxidase (0.1 mL) were added. The change in absorbance over 1 min was recorded and the concentration of xanthine oxidase was adjusted to obtain a linear curve with a slope of 0.024 - 0.026 units of absorbance per min. Potato tissue extract was added instead of the phosphate buffer and the shift in absorbance over 1 min was monitored. One unit of SOD activity was defined as the amount of protein that inhibited NBT reduction to 50% of the maximum.

### 2.8 Data analysis

Four biological replicates were used for each experimental unit. Mean, standard deviation, and standard errors were calculated using Microsoft Excel tools. The data were analyzed statistically using R software and two-way analysis of variance (ANOVA). Least significant difference (LSD) between main effect of cultivar (C) and treatment (T) and their interaction (cultivar × treatment) was performed by Tukey’s HSD test at 95% confidence level and data of mean square (MS) for all biochemical parameters were presented (**Table 1**).

**Table 1:** Analysis of variance (ANOVA) table indicating mean square (MS) and statistically significant differences between cultivars (C) and wound-healing treatments (T) and their interaction (C × T) for SPP rating, total phenolic (TSP) content, phenolic acid content, proline content, activities of enzymes associated with pentose phosphate pathway, TCA cycle, proline catabolism, and antioxidants at 3 (A), 6 (B), and 9 (C) days after wounding of potato tuber tissues.

	df	SPP Rating	TSP	Ferulic Acid	Chlorogenic Acid	Gallic Acid	Catechin	G6PDH	SDH	PDH	Proline	TA	CAT	GPX	SOD
3 D (A)															
C	4	0.9 n.s.	0.80*	84076*	27288*	181.6*	14.1n.s.	245.6*	1.1*	425.2*	2300.0*	3374.6*	48.0*	1.3*	0.3 n.s.
T	3	16.0*	0.04 n.s.	50017*	164924*	40.1 n.s.	244.1*	8.75 n.s.	0.1 n.s.	60.4 n.s.	116.4 n.s.	592.9*	49.0*	0.07 n.s.	0.8*
C × T	12	2.8*	0.1*	27414*	44637*	46.9*	54.7*	54.1*	0.2*	104.0*	552.7*	826.2*	21.2*	0.3*	0.6*
6 D (B)															
C	4	0.5 n.s.	0.3*	45605*	64548*	118.3*	190.8*	136.1*	0.3*	253.8*	1326.5*	1187.2*	17.2 n.s.	0.5*	0.2 n.s.
T	3	14.5*	0.3*	101026*	141757*	25.7 n.s.	44.8 n.s.	61.2*	0.03 n.s.	113.6*	294.5 n.s.	691.3*	116.3*	0.1*	0.6*
C × T	12	2.5*	0.1*	26955*	43238*	32.1*	54.9*	49.6*	0.1*	83.8*	432.8 n.s.	438.2*	25.8*	0.1*	0.3*
9 D (C)															
C	4	0.8 n.s.	0.60*	44996*	11834*	58.7*	93.2n.s.	73.4*	0.1*	77.9*	3836.6*	1216.0*	173.0*	0.1*	n.d.
T	3	19.1*	0.12 n.s.	38521*	182176*	139.7*	295.4*	81.8*	0.01 n.s.	26.1 n.s.	350.5 n.s.	257.9*	115.7*	0.1*	n.d.
C × T	12	3.3*	0.16*	16573*	34276*	37.6*	97.9*	36.2*	0.1*	30.5*	1054.9*	356.6*	65.9*	0.1*	n.d.

*Significant at p ≤ 0.05 based on Tukey’s test.

n.s., not significant.

n.d., not detected.

## 3 Results and discussion

### 3.1 Histological determination of the accumulation of SPP in wounded surface of potato tubers

**Figure 2:**
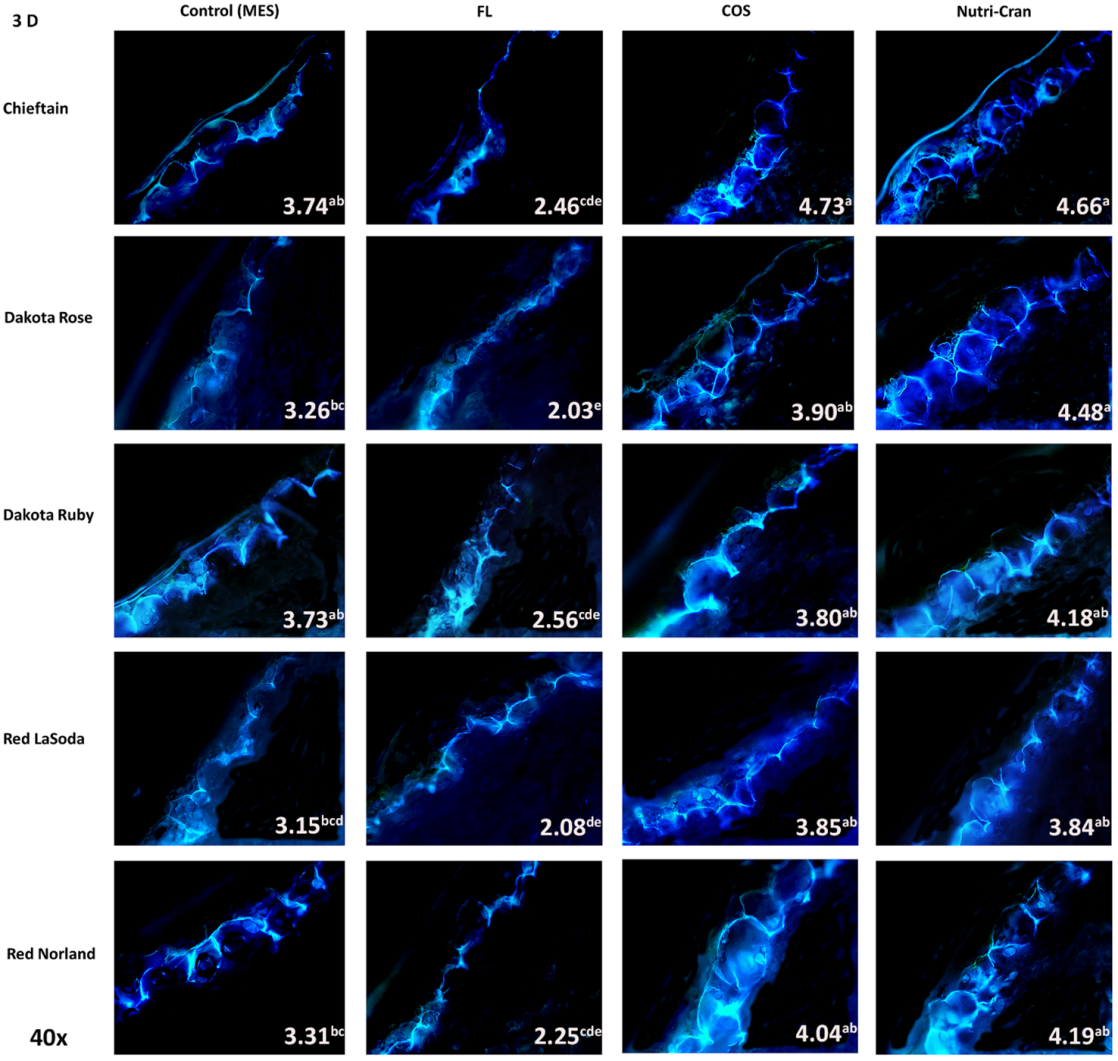
Formation of suberin polyphenolics (SPP) at the first tangential cell layer of the wounded surface of potato tuber tissues (cvs. Chieftain, Dakota Rose, Dakota Ruby, Red LaSoda, and Red Norland). Picture depicts the variations in the SPP formation (40x magnification) with different treatments (Control, fluridone-FL, chitosan oligosaccharide-COS, and cranberry pomace residue- Nutri-Cran) at 3 d after wounding. Data indicates the SPP rating based on the scale developed by Lulai and Corsini, 1998. At 3 d (A), contiguous SPP formation was noticed in all potato cultivars, while with elicitor treatments (COS and Nutri-Cran) SPP accumulation expanded to 50-80% of the first cell layer. Different lowercase letters represent statistically significant differences between cultivar × treatment interaction based on Tukey’s HSD test at 95% confidence level.

**Figure 3:**
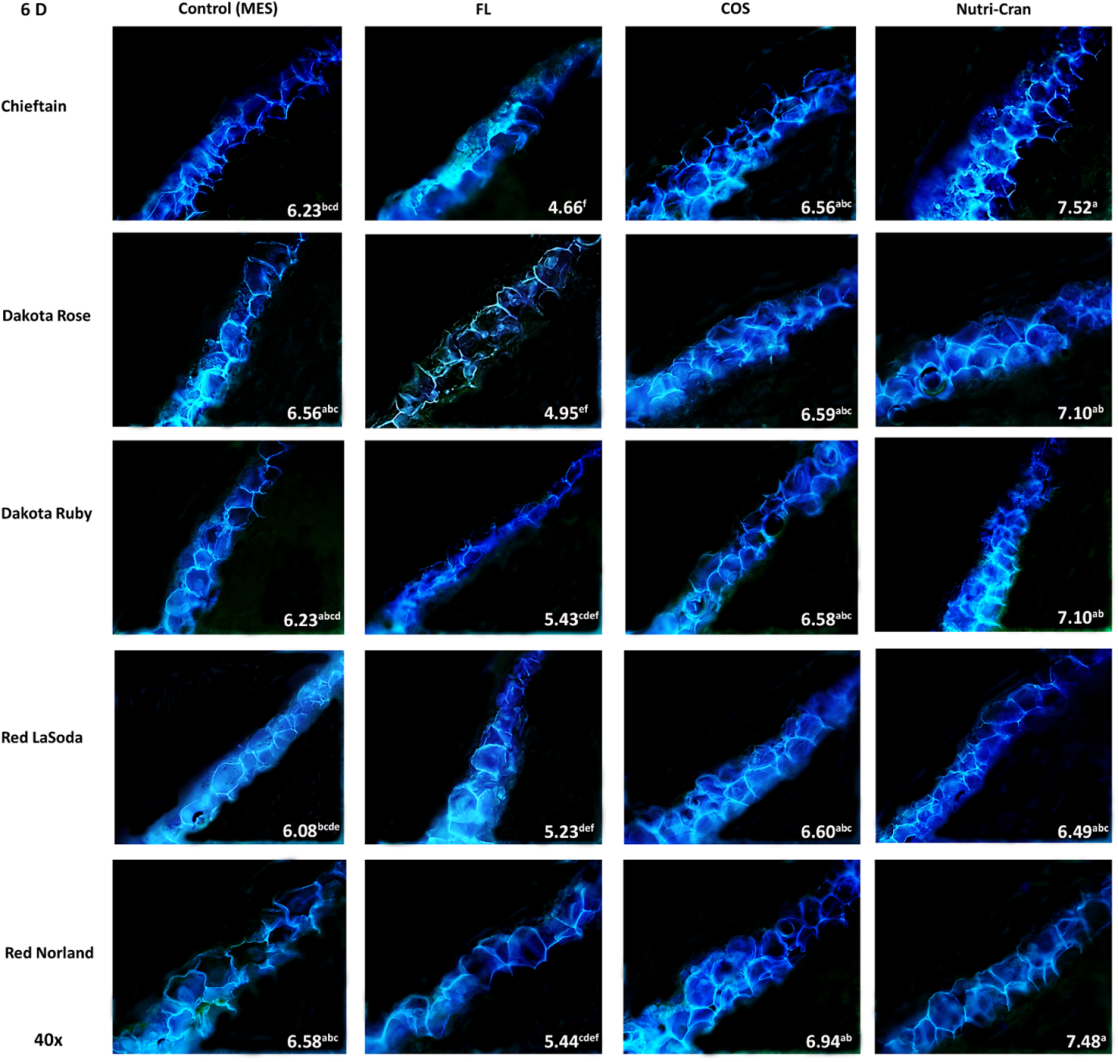
Formation of SPP at the wounded surface of potato tuber tissues (cvs. Chieftain, Dakota Rose, Dakota Ruby, Red LaSoda, and Red Norland). Picture depicts the variations in the SPP formation (40x magnification) with different treatments (Control, fluridone-FL, chitosan oligosaccharide-COS, and cranberry pomace residue- Nutri-Cran) at 6 d after wounding. At 6 d (B), SPP accumulation completed the first cell layer in control sample and spread to second cell layers with elicitor treatments. In Chieftain, Dakota Ruby, and Dakota Rose, accumulation of SPP was not even complete at first cell layer with FL treatment, indicating a significant inhibition. Different lowercase letters represent statistically significant differences between cultivar × treatment interaction based on Tukey’s HSD test at 95% confidence level.

**Figure 4:**
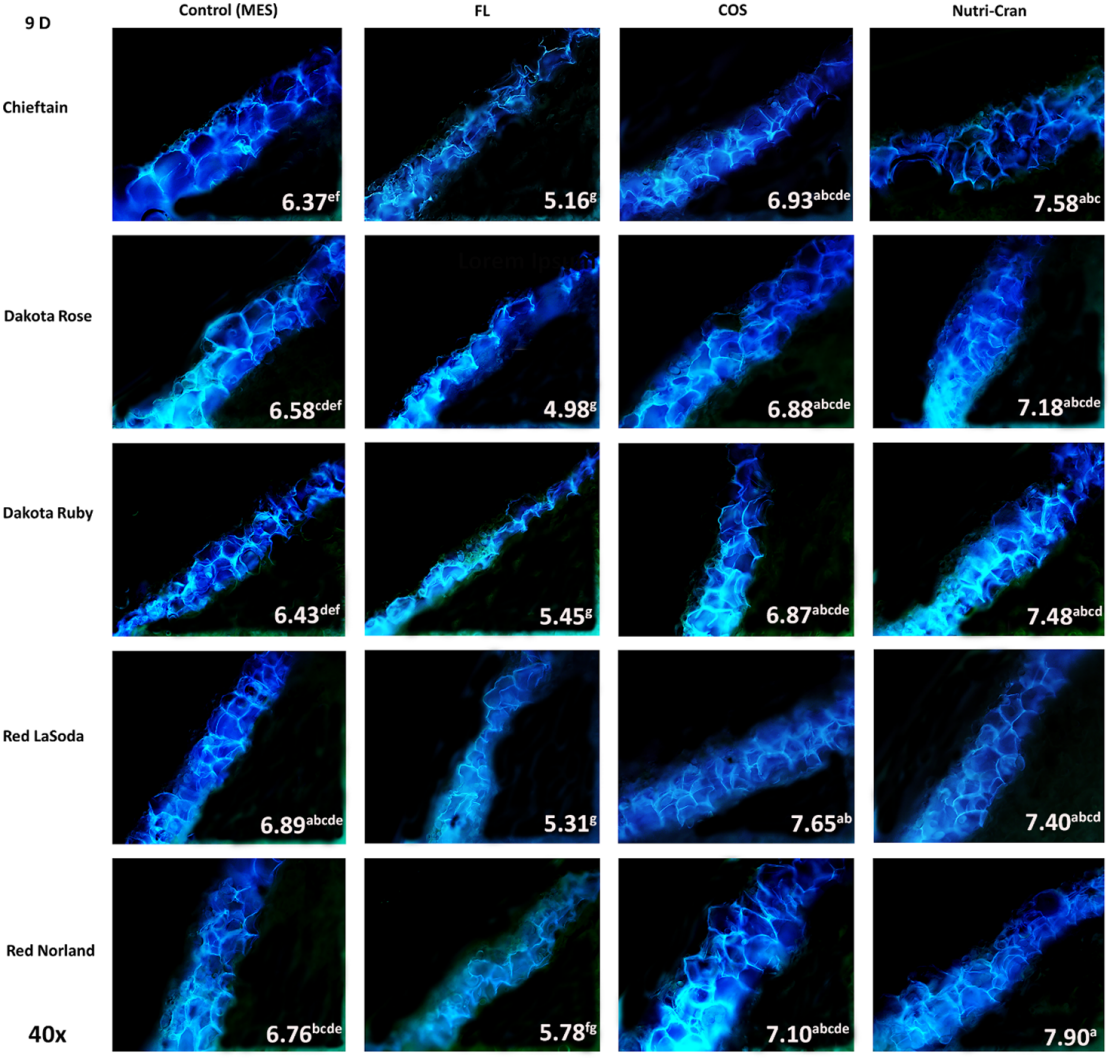
Formation of SPP at the wounded surface of potato tuber tissues (cvs. Chieftain, Dakota Rose, Dakota Ruby, Red LaSoda, and Red Norland). Picture depicts the variations in the SPP formation (40x magnification) with different treatments (Control, fluridone-FL, chitosan oligosaccharide-COS, and cranberry pomace residue- Nutri-Cran) at 9 d after wounding. Completion of SPP accumulation at second cell layer was observed at 9 d (C) in control sample, while elicitor treatment enhanced SPP accumulation to third cell layer in most cultivars. Different lowercase letters represent statistically significant differences between cultivar × treatment interaction based on Tukey’s HSD test at 95% confidence level.

Formation of closing layer in existing parenchyma cells at the wounded site, which is also known as primary suberization is the initial WH response of potato tuber and it generally occurs within 0-8 days after wounding (Lulai and Neubauer, 2014). At time of wounding, cut surface of potato tuber tissues do not possess any suberin biopolymer and deposition of SPP only initiate during the healing process. Based on this critical time period of closing layer formation, SPP accumulation at wounded surface of tuber tissues were determined at 3, 6, and 9 d after wounding. At day 3, contiguous accumulation of SPP was observed at outer cell layer of all wounded tuber tissues (average SPP rating of 3.5) with chieftain having greater average SPP rating (3.9) and Red LaSoda having the lowest average SPP rating (3.2) (**Figure 2**). However, at 6 and 9 d, higher SPP rating was observed in Red Norland tuber tissues (**Figures 3**, **4**). In a study using similar tuber wounding model, Kumar et al. (2010) and Chintha et al. (2023) reported suberization rating of 3.0-4.0 after 3 and 4 d of wounding. Except for potato cultivar Red LaSoda, greater SPP rating was observed in Nutri-Cran treated tissues and it was statistically significant (*p ≤* 0.05) for Dakota Rose at 3 d, Chieftain at 6 d and for Red Norland and Chieftain at 9 d, when compared to the control (MES) treatment (**Figures 2**-**4**). For Red LaSoda, COS elicitor treated tissues exhibited higher SPP rating. Average SPP rating of 7.0-7.5 after 9 d of wounding indicated complete formation of SPP at first two cell layers of the wounded surface and its expansion towards third cell layer. Lulai and Neubauer (2014) also reported similar SPP rating in wounded potato tuber tissues after 7-8 d of wounding. Previously, Chintha et al. (2023) observed improvement in SPP rating in certified mini-tubers of cvs. Russet Burbank and Russet Norkotah with COS elicitor treatment and after 4 d of wounding. Similarly, Xie et al. (2023) reported enhanced deposition of SPP and lignin in wounded tuber tissues with COS elicitor treatment.

Overall, FL treatment significantly reduced SPP accumulation at all time points and in all five potato cultivars (**Figures 2**-**4**). Previous studies suggested that ABA is directly involved in wound-induced suberization process of potato tubers and inhibition of ABA biosynthesis by FL can reduce SPP formation (Lulai and Suttle, 2004; Lulai et al., 2008). In our study, steep changes in SPP rating were observed between 0-6 d, while it slowed down between 6-9 d, indicating plateauing of SPP deposition at the end of the closing layer formation. Yang et al. (2020) reported 13 to 36% improvement in SPP formation in wounded potato tuber tissues (cv. Longshu No. 7) after 7 d of wounding and with hot water dipping treatment. Similarly, Han et al. (2022b) reported improvement in SPP accumulation after 7 days of wounding of potato tuber tissues (cv. Atlantic) with BRs treatment. In our results, 8-37% improvements in SPP formation were observed with Nutri-Cran treatment. However, response to elicitor treatments were specific to cultivars, as not all cultivars had similar rate of improvements in SPP formation. Red Norland is considered having better skin set when compared to Dakota Ruby and Dakota Rose. Skin set capabilities of these cultivars also seem to align with their WH responses. Therefore, improvement in SPP formation in wounded tissues of Dakota Rose with Nutri-Cran treatment has greater relevance for enhancing WH responses of cultivars known to heal poorly. Future studies with other agronomically relevant potato cultivars will also help to confirm the potential link between the WH and skin set traits observed in this study.

### 3.2 Wounding and elicitor induced changes in TSP content and phenolic profile

**Figure 5:**
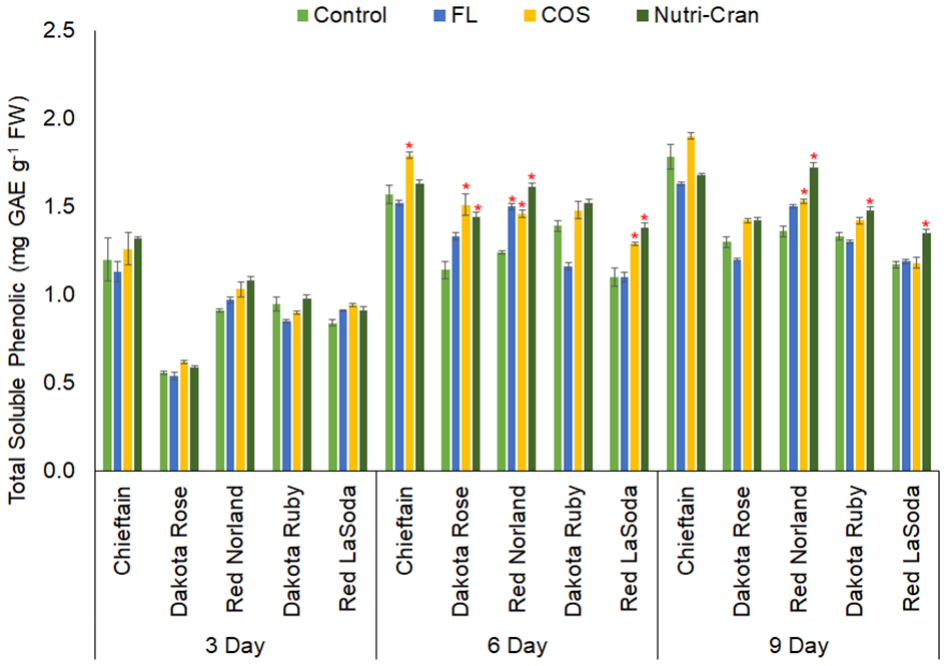
Total soluble phenolic (TSP) content (mg gallic acid equivalent g^-1^ fresh weight) in elicitor treated tuber tissues of five red skin potato cultivars at 3, 6, and 9 d after wounding. Vertical bar represents standard error and * indicates statistically significant differences between control and treatments based on Tukey’s HSD test at 95% confidence level.

Phenolic acids play an integral role in the make-up of SPP domain and matrix of the suberin layer in wounded surface of the potato tuber tissues (Lulai and Neubauer, 2014). Wound-induced accumulation of phenolics in potato tuber tissues was widely reported by previous studies (Reyes and Cisneros-Zevallos, 2003; Dastmalchi et al., 2014; Jiang et al., 2019; Xie et al., 2023). In this study, significant (*p ≤* 0.05) differences in TSP content among potato cultivars and cultivar × treatment interactions were observed at all sampling time points (**Table 1**, **Figure 5**). Irrespective of elicitor treatments, rapid increase in TSP content was observed between 0- 6 d after wounding (**Figure 5**). Additionally, significantly greater TSP content was observed in wounded tissues of Chieftain followed by Red Norland, when compared to other three potato cultivars. Interestingly, Chieftain and Red Norland tubers were more tolerant to wounding stress and had comparatively higher SPP ratings in our study. Previously, Dastmalchi et al. (2019) reported presence of phenolic acid biomarkers in WH tissues of potato tubers (cvs. Russet Norkotah, Atlantic, Chipeta, and Yukon Gold) at early WH stage (0-1 d after wounding). Therefore, TSP content and specific phenolic acid composition can be used as potential biomarker to determine WH trait of some potato cultivars.

Elicitor treatments also enhanced TSP content in wounded potato tuber tissues, but it was specific to the cultivars. At 6 d, both elicitor treatments (COS and Nutri-Cran) resulted into significantly higher TSP content in Red Norland, Dakota Rose, and Red LaSoda, while COS treatment only enhanced TSP content in wounded tuber tissues of Chieftain (**Figure 5**). Even at 9 d, Nutri-Cran treatment increased TSP content in wounded tuber tissues of Red Norland, Dakota Ruby and Red LaSoda. Additionally, COS treatment increased TSP content in Red Norland tuber tissues at 9 d after wounding. Previously, enhanced phenolic content was observed with COS elicitor treatments in wounded potato tuber tissues (Chintha et al., 2023; Xie et al., 2023). Xie et al. (2023) also observed stimulation in biosynthesis of cinnamic, caffeic, *p*-coumaric, and ferulic acids with COS elicitor treatment in potato tuber tissues.

**Figure 6:**
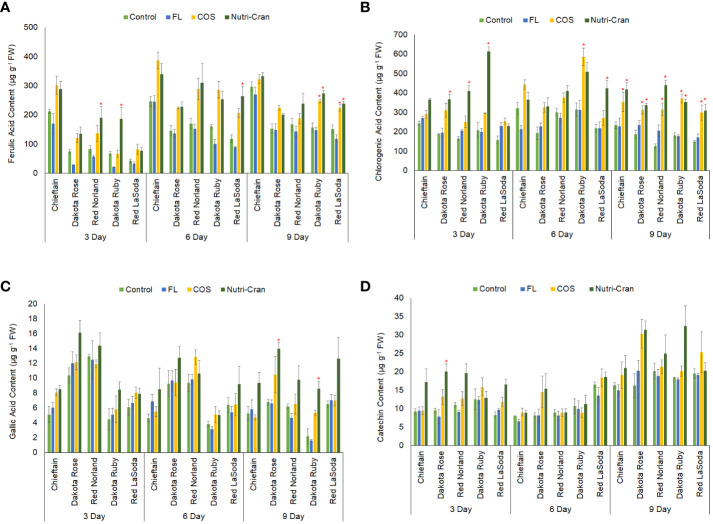
Ferulic acid **(A)**, chlorogenic acid **(B)**, gallic acid **(C)**, and catechin **(D)** content (μg g^-1^ fresh weight) in elicitor treated tuber tissues of five red skin potato cultivars at 3, 6, and 9 d after wounding. Vertical bar represents standard error and * indicates statistically significant differences between control and treatments based on Tukey’s HSD test at 95% confidence level.

In our results, chlorogenic acid, ferulic acid, gallic acid, and catechin were detected in wounded potato tuber tissues (**Figure 6**). Chlorogenic and ferulic acids are hydroxycinnamic acid group compounds, which form the polyaromatic domain of the suberin layer (Bernards et al., 1995; Lulai, 2007). Immediately after wounding (0 h), no ferulic acid was detected in tuber tissue samples, while chlorogenic acid content was quantified in negligible amount. However, between 0 to 3 d, significant increase in ferulic and chlorogenic acid content was observed in wounded tissues of all five cultivars, indicating wound-induced accumulation of these hydroxycinnamic acid group of phenolic compounds. Similar to the results of the TSP content, higher ferulic acid was detected in Chieftain and Red Norland tuber tissues at all time points (**Figure 6A**). At 3 d, Nutri-Cran treatment significantly enhanced ferulic acid content in Red Norland and Dakota Ruby tuber tissues, while FL treatment significantly reduced ferulic acid content in all cultivars. Nutri-Cran treatment also increased ferulic acid content in Red LaSoda tuber tissues at 6 and 9 d and in Dakota Ruby at 9 d. However, COS treatment only resulted into greater ferulic acid content in Dakota Ruby at 9 d. Most significant impact with Nutri-Cran treatment was observed on chlorogenic acid content, as it increased content of this phenolic acid in wounded tissues of 3 cultivars at 3 d (except Red LaSoda and Chieftain) and all five cultivars at 9 d (**Figure 6B**). While COS treatment also increased chlorogenic acid content of wounded tissues of Dakota Ruby at 6 and all cultivars at 9 d. The increased chlorogenic and ferulic acid content in wounded tuber tissues with Nutri-Cran and COS treatments might have relevance in improving SPP formation and associated WH response of potato tubers. Previously, Ramamurthy et al. (1992) reported many folds increase in chlorogenic, caffeic, and ferulic acid content during WH of potato tubers. Wounding-induced accumulation of chlorogenic acid and chlorogenic acid isomers were also found in potato tubers, particularly at higher temperature (20°C), which favors WH process (Torres-Contreras and Jacobo-Velázquez, 2021). In our study, Nutri-Cran treatment also enhanced gallic acid (Dakota Rose and Dakota Ruby) and catechin content (Dakota Rose) (**Figures 6C, D**). Overall, results of phenolic acid profile indicated that elicitor treatments, particularly Nutri-Cran has positive impact on accumulation of phenolics such as ferulic and chlorogenic acids that are critical for WH of potato tubers. Biosynthesis of phenolics and phenylpropanoid metabolism in plant tissues are also associated with regulation of primary metabolism and distribution of carbon flux through major metabolic pathways such as oxidative PPP, shikimate, glycolysis, and TCA cycle (Shetty and Wahlqvist, 2004). Therefore, determining activities of key enzymes and metabolites in these major pathways also sheds light on potential metabolic regulations in wounded plant tissues and how it influences biosynthesis of secondary metabolites and suberin compounds.

### 3.3 Wounding and elicitor induced changes in G6PDH, SDH, and PDH activities and proline content in potato tuber tissues

Wounding stress not only induces stress signaling and accumulation of stress protective phenolic metabolites, but also alter primary carbon metabolism and respiration rate in plant tissues (Lafta and Fugate, 2011). Increased rate of oxidative PPP and TCA cycle, along with upregulation of phenylpropanoid metabolism was previously observed in fresh cut white mushroom slices (*Agaricus bisporus)* (Wang et al., 2022). Oxidative PPP provides precursors to downstream pathways such as shikimate pathway and play an important role in biosynthesis of secondary metabolites including phenolics in potato tuber tissues during WH responses (Woolfson et al., 2023). Previously, Chintha et al. (2023) observed enhanced activity of G6PDH, a key rate limiting enzyme of oxidative PPP, in wounded and COS treated potato tuber tissues. Activation of G6PDH is also reported during WH of hot water treated carrot (*Daucas carota* L.) (Jiang et al., 2024). Upregulation of PPP is associated with antioxidant enzyme responses and phytohormone regulation, both are critical for WH of plant tissues.

**Figure 7:**
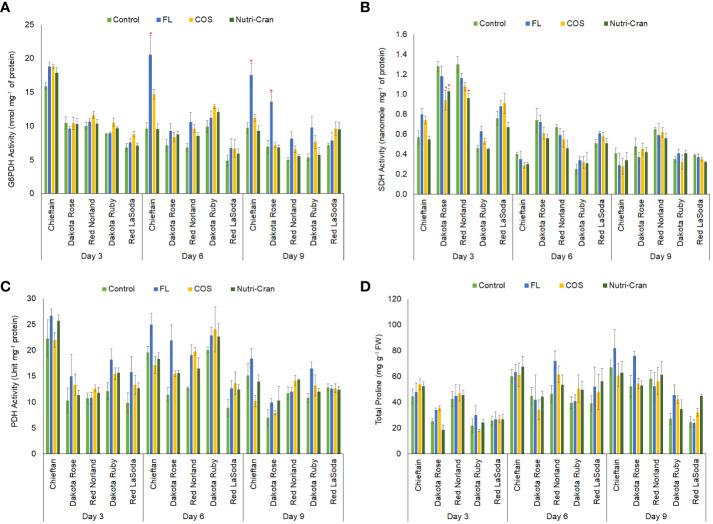
Activities of glucose-6-phosphate dehydrogenase **(A)** (nanomole mg^-1^ of protein), succinate dehydrogenase **(B)** (nanomole mg^-1^ of protein), proline dehydrogenase **(C)** (unit mg^-1^ of protein) and total proline **(D)** content (mg g^-1^ fresh weight) in elicitor treated tuber tissues of five red skin potato cultivars at 3, 6, and 9 d after wounding. Vertical bar represents standard error and * indicates statistically significant differences between control and treatments based on Tukey’s HSD test at 95% confidence level.

In this study, significantly higher G6PDH activity was observed in wounded tissues of Chieftain (**Figure 7A**), which also exhibited higher SPP ratings (**Figures 2**-**4**). Interestingly, greater G6PDH activity was noticed with FL treatment. Inhibition of ABA biosynthesis by FL might have promoted G6PDH activity in wounded potato tubers. These results align with reports suggesting ABA can negatively contribute to oxidative PPP regulation by inhibiting G6PDH activity (Swamy and Sandhyarani, 1986; Gude et al., 1988; Yang et al., 2019). However, higher G6PDH activity did not translate into greater SPP formation in FL treated tissues as PPP regulation could not be able to compensate the critical role of ABA in WH responses of potato tubers. In this study, both elicitor treatments did not increase G6PDH activity in wounded potato tuber tissues.

To understand the respiratory rate, role of TCA cycle, and energy metabolism during WH of potato tubers, we determined the activity SDH, a key enzyme of the TCA cycle. Greater SDH activity was observed in wounded tissues of Red Norland and Dakota Rose tubers (**Figure 7B**). However, Nutri-Cran treatment reduced SDH activity in both cultivars at 3 d when compared to the control treatment. Except for Dakota Ruby, wounded tissues from control (MES) and FL treatments had higher SDH activity. Therefore, elicitor treatment might have suppressed the activity of SDH and altered the carbon and energy metabolism in select cultivars during WH processes of potato tubers. Energy conservation along with upregulation of stress protective metabolic responses are critical for WH and shelf-life of perishable fruits and vegetables during postharvest storage. Jiang et al. (2022) suggested that WH of potato tubers can reduce respiration rate to preserve energy and to extend shelf-life. TCA cycle also provides precursors for synthesis of proline, another important biomarker of plant’s stress response regulation.

In this study, higher PDH activity and proline content were observed in wounded tissues of Chieftain potato cultivar (**Figures 7C, D**), which exhibited rapid formation of SPP. Average proline content also increased from 0 d to 6 d in all potato cultivars, indicating a wound-induced accumulation of this stress-response related amino acid in potato tubers (**Figure 7D**). However, elicitor treatments did not significantly alter the proline content in wounded potato tuber tissues during the closing layer formation. Previously, Liu et al. (2022) reported that exogenous application of proline on whole tubers (pre-treatment) before harvest reduced the enzymatic browning and polyphenolic oxidase (PPO) activity in sliced potato tubers after harvest. However, this proline pre-treatment also suppressed phenolic content in sliced potato tubers. Collectively these results suggest that proline accumulation takes place following wounding, but it might not be an essential component in phenolic biosynthesis and SPP formation in potato tuber tissues. We also measured the activity of PDH, a key enzyme that is involved in proline catabolism and plays role in oxidative stress regulations in plants (Servet et al., 2012). Similar to the trend of proline content and G6PDH activity, higher PDH activity was observed in wounded tissues of Chieftain at all time points and in Dakota Ruby at 6 and 9 d (**Figure 7C**). No significant differences in PDH activity between treatments were observed in this study, indicating that proline and proline catabolism did not play significant role in tuber WH processes in this study; especially elicitor treatments did not influence proline metabolism.

### 3.4 Wounding and elicitor induced changes in total antioxidant activity and activities of major antioxidant enzymes (CAT, GPX, SOD)

**Figure 8:**
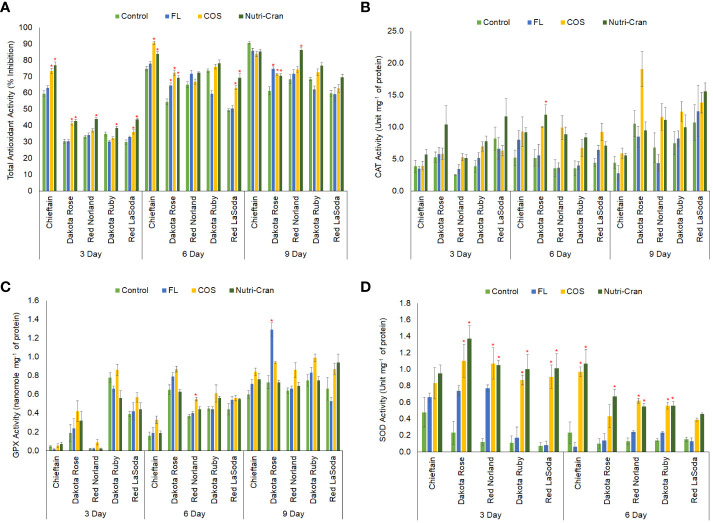
Total antioxidant activity (ABTS-based % inhibition) **(A)** and activities of catalase **(B)** (unit mg^-1^ of protein), guaiacol peroxidase **(C)** (nanomole mg^-1^ of protein), and superoxide dismutase **(D)** (unit mg^-1^ of protein) in elicitor treated tuber tissues of five red skin potato cultivars at 3, 6, and 9 d (no SOD activity at 9 d) after wounding. Vertical bar represents standard error and * indicates statistically significant differences between control and treatments based on Tukey’s HSD test at 95% confidence level.

Oxidative stress regulation and ROS mediated signaling plays an important role in WH of biological tissues including potato tuber tissues (Dastmalchi et al., 2015; Jiang et al., 2020; Yang et al., 2020; Zhou et al., 2024). Enzymatic and non-enzymatic antioxidants counter the over generation of ROS and stabilize the cellular redox regulation, which is important for tissue integrity and repair during WH process. In this study, significantly higher antioxidant activity was observed in Chieftain at all time points (**Figure 8A**). Furthermore, Nutri-Cran and COS treatments significantly (*p ≤* 0.05) enhanced total antioxidant activity in wounded tissues of potato cultivars, particularly at 3 and 6 d. At 3 d, except for Dakota Ruby, Nutri-Cran treatment increased total antioxidant activity in other four cultivars, while COS treatment enhanced total antioxidant activity in wounded tissues of Chieftain, Dakota Rose, and Red LaSoda. Similarly, greater antioxidant activity was also observed at 6 d with both treatments in Chieftain, Dakota Rose, and Red LaSoda, while Nutri-Cran increased antioxidant activity in Red Norland at 9 d. Results of the total antioxidant activity corroborated with TSP and phenolic acid results, suggesting that phenolic metabolites and oxidative stress regulation play key roles in WH response of potato tuber tissues.

Furthermore, we also measured the activities of select antioxidant enzymes (CAT, GPX, and SOD) to better understand the specific role of individual antioxidant enzyme in tuber WH responses. Previously, Xie et al. (2023) reported COS induced increase in total antioxidant activity, and activities of CAT, and peroxidase during WH of potato tubers. Exogenous application of CAT inhibitor (aminotriazole) reduced deposition of polyphenolic domain and enhanced accumulation of H_2_O_2_ in wounded potato tuber tissues (cvs. Mérit, Désirée and Gasoré) (Bajji et al., 2007), indicating an important role of CAT in tuber WH process. In this study greater CAT activity was observed in wounded tissues of Dakota Rose and Red LaSoda (**Figure 8B**). Furthermore, Nutri-Cran treatment enhanced CAT activity in Dakota Rose at 6 d. However, COS treatment did not induce CAT activity in this study. CAT helps decomposition of H_2_O_2_ and maintaining redox balance to support repair of cell wall during healing of plant tissues. Results of our study indicated that CAT is important during potato tuber WH. However, cultivar differences played significant role in their response to elicitor treatments.

The activity results of another important antioxidant enzyme (GPX) revealed an interesting trend. No GPX activity was observed in 0 h wounded tissue sample, while GPX activity increased rapidly from 3 to 6 d in most potato cultivars studied (**Figure 8C**). This result suggests that the GPX enzyme activity is wound induced response in potato tuber tissues. Significant differences in GPX activity between cultivars were observed, with Dakota Rose and Dakota Ruby having higher baseline GPX activity. Previously, Zheng et al. (2020) elucidated that peroxidase activity is differentially expressed among different potato cultivars, and peroxidase plays important role in WH response of potato tubers. Overall, COS elicitor treatment slightly enhanced the GPX activity, but it was not statistically significant in most cultivars and at any time points examined. Chintha et al. (2023) reported enhanced GPX activity with COS treatment during WH of Russet Burbank tubers. Whether the impact of elicitor treatments on GPX activity is cultivar specific or not needs further evaluation.

Contrary to the trend of GPX activity, SOD activity gradually decreased from 0 h to 6 d, and no SOD activity was detected in any potato tissue samples at 9 d (**Figure 8D**). However, Nutri-Cran treatment significantly enhanced SOD activity, except for Chieftain at 3 d and for Red LaSoda at 6 d. Similarly, COS treatment also increased SOD activity in wounded tissues of 4 cultivars (Dakota Rose, Dakota Ruby, Red LaSoda, and Red Norland) at 3 d, and in 3 cultivars (Chieftain, Dakota Ruby, and Red Norland) at 6 d. Lin et al. (2011) previously proposed that wounding-induced SOD production helps to quench H_2_O_2_ and reduce the cell death caused by wounding. Zhou et al. (2024) observed that ascorbic acid treatment reduced SOD activity and slowed down WH response in fresh cut potato (cv. Youjin ‘885′). Our results indicated that SOD was required at early stages of WH, but once tissues started to heal, SOD might not be needed to maintain redox balance. Furthermore, enhanced SOD activity with elicitor treatment, might have relevance in improving WH responses of potato tuber tissues, especially immediately after wounding. Collectively, these results suggest that antioxidant enzymes play key regulatory role in WH processes of potato tubers and elicitors can enhance antioxidant enzyme activity to maintain redox homeostasis during healing of potato tuber tissues. Determination of ROS generation in wounded tuber tissues need to be investigated in future studies to reveal the role of redox equilibrium in closing layer formation of potato tuber.

## 4 Conclusion

Results of this wound healing research indicated an improvement in SPP formation with elicitor treatments. Particularly, Nutri-Cran treatment enhanced SPP formation in wounded tissues of Chieftain, Dakota Ruby, and Red Norland, while COS treatment induced same response in Red LaSoda. Additionally, enhanced accumulation of ferulic and chlorogenic acids, which are critical building blocks of suberin biopolymer domain was observed with Nutri-Cran treatment in same cultivars with greater SPP ratings. Elicitor-induced improvement in antioxidant responses, particularly SOD and total antioxidant activity, was also observed in wounded tuber tissues. However, the response to elicitor treatments varied significantly among potato cultivars, with greater improvement of WH response in Chieftain and Red Norland. Biochemical analyses indicated that both elicitor treatments induce stress responses associated with phenolic metabolites and antioxidant enzymes in wounded tissues. Further optimization of these elicitor treatment applications is needed for integration of such treatments in commercial potato postharvest storage facilities for mitigation of wound-related tuber losses.

## Data availability statement

The original contributions presented in the study are included in the article/supplementary material. Further inquiries can be directed to the corresponding author.

## Author contributions

MD: Conceptualization, Data curation, Formal analysis, Funding acquisition, Investigation, Methodology, Project administration, Resources, Software, Supervision, Validation, Visualization, Writing – original draft, Writing – review & editing. DS: Data curation, Formal analysis, Investigation, Methodology, Validation, Visualization, Writing – original draft, Writing – review & editing. FF: Investigation, Methodology, Writing – original draft, Writing – review & editing. KS: Investigation, Writing – original draft, Writing – review & editing. KF: Conceptualization, Writing – original draft, Writing – review & editing.
